# Green Tea Catechin (-)-Epigallocatechin-3-Gallate (EGCG) Facilitates Fracture Healing

**DOI:** 10.3390/biom10040620

**Published:** 2020-04-16

**Authors:** Sung-Yen Lin, Jung Yu Kan, Cheng-Chang Lu, Han Hsiang Huang, Tsung-Lin Cheng, Hsuan-Ti Huang, Cheng-Jung Ho, Tien-Ching Lee, Shu-Chun Chuang, Yi-Shan Lin, Lin Kang, Chung-Hwan Chen

**Affiliations:** 1 Orthopaedic Research Center, Kaohsiung Medical University, Kaohsiung 80708, Taiwan; tony8501031@gmail.com (S.-Y.L.); cclu0880330@gmail.com (C.-C.L.); junglecc@gmail.com (T.-L.C.); hthuang@kmu.edu.tw (H.-T.H.); rick_free@mail2000.com.tw (C.-J.H.); tn916943@gmail.com (T.-C.L.); hawayana@gmail.com (S.-C.C.); 327lin@gmail.com (Y.-S.L.); 2 Department of Orthopedics, Kaohsiung Medical University Hospital, Kaohsiung Medical University, Kaohsiung 80708, Taiwan; 3 Departments of Orthopedics, College of Medicine, Kaohsiung Medical University, Kaohsiung 80708, Taiwan; 4 Department of Orthopedics, Kaohsiung Municipal Ta-Tung Hospital, Kaohsiung City 80145, Taiwan; 5 Regeneration Medicine and Cell Therapy Research Center, Kaohsiung Medical University, Kaohsiung 80708, Taiwan; 6 Graduate Institute of Medicine, College of Medicine, Kaohsiung Medical University, Kaohsiung 80708, Taiwan; kan890043@gmail.com; 7 Division of Breast Surgery, Department of Surgery, Kaohsiung Medical University Hospital, Kaohsiung 80708, Taiwan; 8 Department of Orthopedics, Kaohsiung Municipal Siaogang Hospital, Kaohsiung Medical University, Kaohsiung 80708, Taiwan; 9 Department of Veterinary Medicine, National Chiayi University, Chiayi City 60054, Taiwan; hhuang@mail.ncyu.edu.tw; 10 Department of Physiology, College of Medicine, Kaohsiung Medical University, Kaohsiung 80708, Taiwan; 11 Department of Obstetrics and Gynecology, National Cheng Kung University Hospital, College of Medicine, National Cheng Kung University, Tainan 70101, Taiwan; 12 Institute of Medical Science and Technology, National Sun Yat-Sen University, Kaohsiung 80424, Taiwan

**Keywords:** (-)-epigallocatechin-3-gallate (EGCG), bone morphogenetic protein-2 (BMP-2), catethin, fracture healing, local use

## Abstract

Green tea drinking can ameliorate postmenopausal osteoporosis by increasing the bone mineral density. (-)-Epigallocatechin-3-gallate (EGCG), the abundant and active compound of tea catechin, was proven to be able to reduce bone loss and ameliorate microarchitecture in female ovariectomized rats. EGCG can also enhance the osteogenic differentiation of murine bone marrow mesenchymal stem cells and inhibit the osteoclastogenesis in RAW264.7 cells by modulation of the receptor activator of nuclear factor-kB (RANK)/RANK ligand (RANKL)/osteoprotegrin (OPG) (RANK/RANKL/OPG) pathway. Our previous study also found that EGCG can promote bone defect healing in the distal femur partially via bone morphogenetic protein-2 (BMP-2). Considering the osteoinduction property of BMP-2, we hypothesized that EGCG could accelerate the bone healing process with an increased expression of BMP-2. In this manuscript, we studied whether the local use of EGCG can facilitate tibial fracture healing. Fifty-six 4-month-old rats were randomly assigned to two groups after being weight-matched: a control group with vehicle treatment (Ctrl) and a study group with 10 µmol/L, 40 µL, EGCG treatment (EGCG). Two days after the operation, the rats were treated daily with EGCG or vehicle by percutaneous local injection for 2 weeks. The application of EGCG enhanced callus formation by increasing the bone volume and subsequently improved the mechanical properties of the tibial bone, including the maximal load, break load, stiffness, and Young’s modulus. The results of the histology and BMP-2 immunohistochemistry staining showed that EGCG treatment accelerated the bone matrix formation and produced a stronger expression of BMP-2. Taken together, this study for the first time demonstrated that local treatment of EGCG can accelerate the fracture healing process at least partly via BMP-2.

## 1. Introduction

Long-bone fracture is a common musculoskeletal trauma representing a considerable burden of disease. Bone fracture healing is a long process usually requiring several months. The delayed union or nonunion rate is approximately 5%–10% [1]. Acceleration the fracture healing process and shortening the recovery time is important. As a result, different types of nourishment and medications have been intensively investigated in order to identify the optimal strategies to promote fracture healing.

The beneficial effects of green tea on bone health have been widely reported. Numerous clinical studies indicated a positive effect between tea consumption and bone health [2]. Those who drink tea habitually have better bone mineral density (BMD) and a lower hip fracture rate [3,4]. Hegarty’s study reported approximately 5% higher lumbar spine BMD in frequent tea drinkers than in those who did not drink tea [5]. A cross-sectional analysis from a 5-year prospective trial related to oral calcium supplements and osteoporotic fractures also demonstrated that tea drinkers had a lower rate of BMD loss compared to non-tea drinkers (1.6% vs. 4% in 4 years) [6]. (-)-Epigallocatechin-3-gallate (EGCG) is widely studied because of its potent antioxidant effects [7]. Previous animal studies found that green tea polyphenol extracts improved several bone loss models related to aging, estrogen-deficiency, and chronic inflammation [8,9,10,11,12,13,14]. Although numerous clinical and animal studies indicated that tea can increase bone volume and diminish osteoporotic fractures, the relationship between tea consumption and fracture healing remained unclear. 

The fracture healing process is a specialized healing process that depends on the coordination of different growth factors to stimulate bone formation. Bone morphogenetic protein-2 (BMP-2) is a powerful osteogenic factor that induces osteoblast differentiation and promotes bone formation [15]. Enhanced BMP-2 expression is known to play an essential role in the initiation of fracture healing [16]. We found that EGCG enhanced BMP-2 mRNA expression in human bone marrow derived mesenchymal stem cells (BMSCs) [17]. EGCG can facilitate osteogenic differentiation of both murine and human BMSCs and eventually increase mineralization in vitro [17,18]. We previously reported that intraperitoneal injection of EGCG for 3 months in ovariectomized rats could increase bone volume and microarchitecture at a dosage of 3.4 mg/kg/day, which achieved 10 µmol/L in serum. The effect of EGCG may rely on BMP-2 [19]. We also found that local treatment of EGCG could improve the healing of femoral bone defects and that this effect might be mediated at least in part by BMP-2 [20]. In this study, we hypothesized that local percutaneous injection of EGCG could promote fracture healing by enhancing BMP-2 expression. 

## 2. Materials and Methods 

### 2.1. Chemicals 

High purity (>99%) grade EGCG (No. E4143) was purchased from Sigma-Aldrich (St. Louis, MO, USA) and was dissolved in dimethylsulfoxide (DMSO).

### 2.2. Experiment Animals

This animal study was approved by the Institutional Animal Care and Use Committee. Male Sprague–Dawley (SD) rats at 12 weeks of age were obtained from the National Laboratory Center (Taipei, Taiwan) with a mean body weight of 350 ± 25 g, and were provided with free food and water in a temperature-controlled room (25 ± 1 °C) and kept on a 12:12 light–dark cycle during the experiment. Fifty-six 4-month-old rats were randomly divided into two groups after being weight-matched: fracture with vehicle treatment as control group (Ctrl) (*n* = 28) and fracture with treatment of EGCG (EGCG) (*n* = 28). We used EGCG, 40 µL at 10 µmol/L, with a total dose of 0.52 µg/kg/time [20]. Isolated right tibia fractures were made with a saw. Percutaneous local injection of either vehicle or EGCG were given locally and daily 2 days after the fracture creation for 2 weeks (*n* = 14 at each group). Micro-computed tomography (μ-CT), biomechanical analysis (*n* = 7 at each group), and histological study (*n* = 7 at each group) were done 2 and 4 weeks after treatment [19,20,21]. 

### 2.3. Tibial Fracture Model 

An isolated right tibia fracture with intramedullary needle fixation was selected as the bone fracture healing model. Each animal was anesthetized with ketamine (60 mg/kg) administered via intraperitoneal injection. The right hind limb was prepped and draped in a sterile manner. A 1 cm longitudinal skin incision was made over the antero-medial aspect of the lower limb. The tibia was exposed without elevating the periosteum, and osteotomized using an oscillating saw under continuous irrigation. After open osteotomy, a 23-gauge syringe needle was inserted into the bone marrow cavity of the tibia to stabilize the fracture. The surgical wound was sutured and covered with sterile dressings for two days. The proper alignment after fixation was radiographically confirmed.

### 2.4. Radiographic and μCT Analyses

During radiologic examination and micro-CT image scanning, the animals were sedated. For investigation of the fracture repair process in living animals, small animal micro-CT (Skyscan 1076, Bruker, Belgium) and image software (CTAn) were used and calculated at the indicated time point (*n* = 14 in each time point). For scanning, the scan settings were an aluminum filter of 0.5 mm, 9 μm scanning resolution, X-ray voltage of 50 kV, X-ray current of 200 mA, and exposure time of 600 ms. The analysis began from the proximal tibia and through the whole fracture site until the distal tibia [19,21,22,23,24,25,26].

### 2.5. Histological Study 

Rats were sacrificed at 2 weeks and 4 weeks for histological analysis after surgery (*n* = 7 in each time point). The bone samples were harvested after sacrifice and fixed with 10% neutral buffered formaldehyde for 2 days, decalcified in 14% ethylenediaminetetraacetic acid (EDTA)/phosphate buffered saline (PBS) for 14 days and then embedded in paraffin. The 5-µm bone sections were hematoxylin and eosin stained for histomorphometry of the bone volume after decalcification. At a magnification of 40×, we defined the counted callus area as the 1-mm regions proximal and distal to the bone graft ends. The area of callus formation and the intact tibia bone was measured using Image-Pro Plus 5.0 software (Media Cybernetics, Inc., Rockville, MD, USA). We calculated the percentage of bone matrix of the callus and the intact tibia and compared with the results from the control group [27,28,29,30]. 

### 2.6. Immunohistochemistry (IHC) of BMP-2

The samples were prepared for indirect immune detection using a rabbit polyclonal anti-BMP-2 (Abcam, Cambridge, MA, USA) and mouse and rabbit specific horseradish peroxidase/ diaminobenzidine detection IHC kit (Abcam, Cambridge, MA, USA) by protocols provided by manufacture. The sections were then counterstained with hematoxylin to visualize cell nuclei. BMP-2 were stained brown [20]. Under high power magnification, the BMP-2 stain in the callus area was measured and quantified [20,29].

### 2.7. Three Point Bending for Biomechanical Testing

Instron 4466 (model 4465; Instron, Canton, Massachusetts) was used for the mechanical property of the repaired fracture in the tibia bone samples after the soft tissues were removed (*n* = 7 in each time point). For three-point bending, the tibia bone was placed between two metal supports, and a single-pronged loading device was applied to the opposite surface at a point precisely in the middle between the two supports. The distance between the two supports was 4 cm. The loading force was 1 N at a rate of 1 mm/min and directed to the mid-diaphyseal region [21]. We measured the deflection of the bone at the point of load application and the simultaneous measurement of the load, yielding a force–deflection graph. The biomechanical parameters, including yield point, maximal load, fracture load, and whole-bone stiffness (defined as the slope of the early, linear portion of the load–deflection curve) were recorded. The Young’s modulus of the material was also measured by the geometry of the loading device and the stiffness of the bone. 

### 2.8. Statistical Analysis 

All data were expressed as mean ± standard error by at least three independent experiments. Comparisons of the data were analyzed by one-way ANOVA, and the Scheffe post hoc test using SPSS (version 22 for Windows, SPSS Inc, Chicago, IL, USA). Statistical significance was defined as *p* < 0.05.

## 3. Results

### 3.1. X-ray and Microarchitecture Assessment by μ-CT

**Figure 1:**
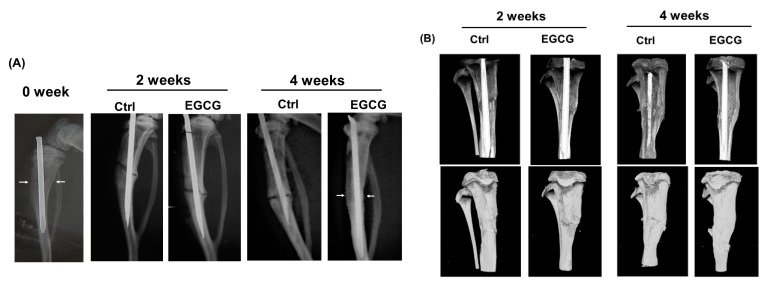
(-)-Epigallocatechin-3-gallate (EGCG) promotion of bone fracture healing in rats. The images of X-ray and micro-computed tomography (μ-CT). (**A**) The X-ray radiographic analysis showed a clear fracture gap at weeks 0, 2, and 4 in the control group, and this fracture was gradually blurred at weeks 2 and 4 in the EGCG treated group. Arrows indicate the fracture site. (**B**) The results of μ-CT analysis also showed a gradual union of fracture gap at the end of weeks 2 and 4 after treatment. Compared to the control group, the fracture was united at the end of week 4 after the EGCG treatment, suggesting that fracture healing process was accelerated by EGCG.

The images of x-ray and μ-CT are revealed in Figure 1. The results of the radiographic analysis showed that the administration of EGCG enhanced bone callus formation. With EGCG treatment, the fracture gap decreased at the end of weeks 2 and 4 after treatment both in the X-ray images (Figure 1A) and in the μ-CT images (Figure 1B). Compared to the control group, the fracture gap decreased gradually in the EGCG treated group at weeks 2 and 4. The quantification results of the callus in μ-CT are shown in Table 1. Furthermore, there was no fracture gap visible at week 4 in the EGCG treated group, confirming the beneficial role of EGCG on the fracture healing process. 

### 3.2. Three-Point Bending Test for the Mechanical Properties of the Bone

**Figure 2:**
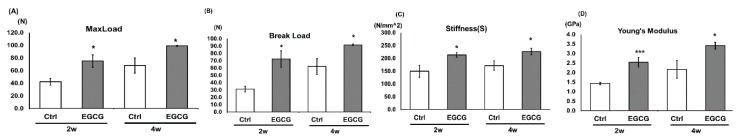
EGCG increased the bone mechanical properties of the fractured tibia. The results of mechanical testing showed that the maximal load (**A**), break load (**B**), stiffness (**C**), and Young’s modulus (**D**) (at weeks 2 and 4) were significantly increased after the treatment of EGCG. All data are expressed as mean ± standard error. * *p* < 0.05 versus control group after treatment. *** *p* < 0.001 versus control after treatment.

After harvesting the tibia, the three-point bending test was used for the mechanical strength of fracture healing. The results of the biomechanical testing showed that the treatment with EGCG enhanced the bone mechanical strength (Figure 2A–D). The maximal load increased from 42.3 ± 5.5 N in the control group to 75.2 ± 9.9 N in the EGCG group at the end of week 2 (*p* < 0.05) and from 68.1 ± 12.0 N in the control group to 99.1 ± 0.7 N in the EGCG group at the end of week 4 (*p* < 0.05) (Figure 2A). The break point improved from 31.2 ± 4.0 N in the control group to 72.2 ± 11.4 N in the EGCG group at the end of week 2 (*p* < 0.05) and from 62.1 ± 11.2 N in the control group to 91.5 ± 1.1 N in the EGCG group at the end of week 4 (*p* < 0.05) (Figure 2B). The stiffness also improved from 149.1 ± 23.9 N/mm^2^ in the control group to 213.5 ± 8.8 N/mm^2^ in the EGCG group at the end of week 2 (*p* < 0.05) and from 171.4 ± 18.4 N/mm^2^ in the control group to 226.6 ± 12.9 N/mm^2^ in the EGCG group at the end of week 4 (*p* < 0.05) (Figure 2C). Young’s modulus improved from 1.4 ± 0.06 GPa in the control group to 2.5 ± 0.25 GPa in the EGCG group at the end of week 2 (*p* < 0.001) and 2.2 ± 0.5 GPa in the control group to 3.4 ± 0.18 GPa in the EGCG group at the end of week 4 (*p* < 0.05) (Figure 2D). 

### 3.3. Histological Study

**Figure 3:**
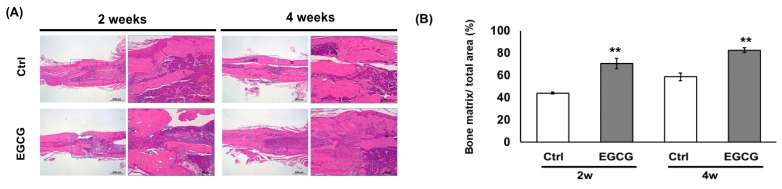
(**A**) Histological analysis. There was significantly more new matrix formation in the EGCG groups than in the control groups. (**B**) The new bone matrix formation in callus tissues was significantly enhanced by EGCG treatment at weeks 2 and 4. ** *p* < 0.01 versus control after treatment.

Histological analysis showed that fracture healing was accelerated by EGCG treatment. More new matrix formation in EGCG groups was noted than that in the control group at the end of both weeks 2 and 4 (Figure 3A). The bone matrix ratio increased from 44.0% ± 0.9% to 70.6% ± 4.7% at the end of week 2 (*p* < 0.01) and 58.6% ± 3.4% to 82.6% ± 7.1% at the end of week 4 (*p* < 0.01) (Figure 3B). The histological study further approved the X-ray and μ-CT results.

### 3.4. IHC Analysis

**Figure 4:**
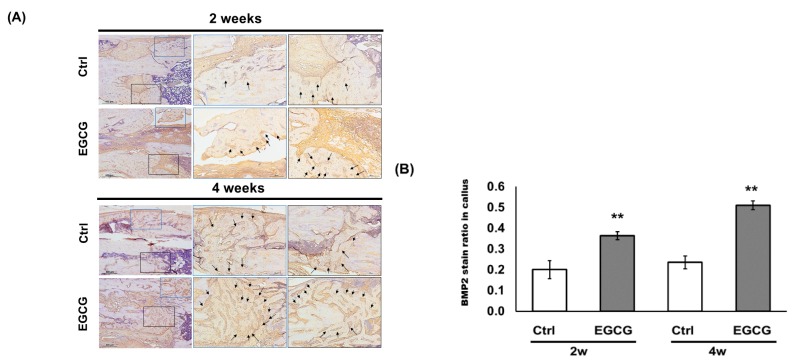
Immunohistochemistry study. (**A**) Immunolocalized bone morphogenetic protein-2 (BMP-2) in new form matrices were stained brown. The arrows indicate positive BMP-2 stains. (**B**) Quantification of immunolocalized BMP-2. EGCG facilitated bone fracture healing by increasing the BMP-2 expression in callus tissue. ** *p* < 0.01 versus control after treatment.

In the IHC study, immunolocalized BMP-2 in new form matrices were quantified (Figure 4A). The brown stained ratio in the callus tissue increased from 0.20 ± 0.04 to 0.36 ± 0.02 and from 0.24 ± 0.03 to 0.51 ± 0.02 at the end of weeks 2 and 4, respectively, with the treatment of EGCG (*p* < 0.01) (Figure 4B). Our findings indicated that EGCG can facilitate bone fracture healing by increasing the BMP-2 expression.

## 4. Discussion

EGCG, one of the most bioactive catechins derived from green tea, can stimulate bone formation both in vitro and in vivo [17,19,20,31]. The effects of EGCG on the promotion of the fracture healing process, however, remain unclear. This is the first study to indicate the beneficial effects of local percutaneous EGCG treatment in facilitating the fracture healing of tibia established by X-ray, µCT images, and eventually mechanical property tests. The study results provided supportive evidence that the local EGCG improved callus formation and increased the bone mechanical strength in the fractured tibia. Significantly, the current study results also indicated that the effect on accelerating fracture healing may be partially related to EGCG’s role in the upregulation of BMP-2.

The healing process of fractured bone is regulated by the differentiation of osteoblasts and BMSCs. In our previous study, we indicated that EGCG can facilitate the osteogenic differentiation of murine and human BMSCs especially at 10 µmol/L [17]. At the same concentration, EGCG can also decrease osteoclastogenesis via the regulation of osteoprotegrin (OPG) and the receptor activator of nuclear factor-kB (RANK) ligand (RANKL) in pre-osteoclast feeder cells, ST2 [32]. Qiu’s study also showed that 10–40 µmol/L of EGCG significantly inhibited hypoxia-induced apoptosis in BMSCs and increased the level of runt-related transcription factor 2 (Runx2), BMP-2, type I procollagen, and alkaline phosphatase activity [33]. We found no published data regarding the effect dose of EGCG on fracture healing. As a result, we chose 10 µmol/L of EGCG in this study. We inferred that EGCG may increase the osteogenic differentiation of BMSCs around the fracture site at this concentration. Moreover, the callus size may be further increased due to decreased osteoclastogenesis.

BMPs are crucial regulators of both chondrocyte and osteoblast differentiation and thus can promote bone formation by improving both intramembranous and endochondral ossification [34]. BMP-2 can strongly induce new bone formation and is one of the most powerful osteoinductive biofactors. BMP-2 can be applied for delayed union, non-union, and bone defects [35]. The role of BMP-2 in fracture healing has been investigated using BMP2 limb bud mesenchyme conditional knockout mice. Researchers demonstrated that the fracture healing process was slower in the heterozygotes compared to the wild-type mice and no fracture healing was noted in the knockout mice [34]. 

The direct application of BMP-2 was also proven to accelerate the healing process in small animal, large animal, and even human clinical trials [36,37,38]. Our previously reported intraperitoneal EGCG use mitigated the BMD decline and ameliorated the bone architecture. Moreover, EGCG treatment also increased the immunolocalized BMP-2 in the bone matrix [19]. In another in vivo study, we locally applied EGCG on femoral bone defects and found that the local use of EGCG at femoral defects could facilitate new bone formation by enhancing the bone volume and subsequently recovered the mechanical strength of the bone. The expression of the immunolocalized BMP-2 was shown to increase up to 3-fold in EGCG treated rats [20]. With this concept, we used EGCG to facilitate tibia fracture healing. Our results revealed that treatment with EGCG, at 10 µmol/L, significantly accelerated the fracture healing and thus improved the mechanical strength of the fractured tibia. 

The modulation of osteoclast activity may be another function of EGCG to increase the callus size. Chen et al. reported that EGCG can inhibit osteoclastogenesis in RAW264.7 cell and ST2 cell co-cultures by modulation of the RANK/RANKL/OPG pathway [32]. Similar results were also reported in Xu’s study, where EGCG and its oxidation product were both found to effectively inhibit RANKL-induced osteoclastogenesis in RAW 264.7 cells [39]. A study by Song et al. also found that EGCG supplementation at a dose of 10 mg/kg/day for 12 weeks markedly increased both BMD and total bone volume and also improved the microarchitecture of the trabecular bone in the femur. 

In an IHC study, researchers reported that EGCG diminished semaphorin 4D expression [40]. Semaphorin 4D is highly expressed in osteoclasts and may potently inhibit bone formation by binding to Plexin-B1 receptors, which are highly expressed by osteoblasts [41]. Semaphorin 4D specific antibody increased osteoblastic bone formation but did not disturb osteoclastic bone resorption and eventually diminished bone loss after ovariectomies [41]. As a result, EGCG could regulate the RANKL-induced osteoclastogenesis and possibly reduce the resorption of woven bones and increase the callus size, which may be another possible mechanism for increased tibia fracture healing.

Previous studies indicated that taking one cup of green tea can achieve a serum level of 1 μmol/L EGCG [42,43]. An oral dose of 1600 mg EGCG can achieved a serum level of 7.6 μmol/L [44]. The effective concentration of EGCG to enhance fracture healing was 10 μmol/L in this study, which can be reached by daily tea consumption. Adequate oral EGCG was used in the study for fracture healing. The molecular mechanisms of EGCG on fracture healing are likely due, at least in part, to the increased BMP-2 expression. Further studies are required to find more molecular mechanisms.

## 5. Conclusions

In conclusion, we found that locally administered EGCG, to a level of 10 μmol/L with 40 μL at a dose of 0.52 µg/kg/time, at a tibia fracture could enhance the fracture healing by improving the callus size and then aid in recovering the mechanical strength of the bone, including the max load, modulus, stiffness, and break load. The promotion effect of EGCG on fracture healing might be in part due to the effects on BMP-2.

## Figures and Tables

**Table 1:** The quantification results of callus in μ-CT.

	Week 2		Week 4	
	Control	EGCG	Control	EGCG
Tissue volume (mm^3^)	440.87 ± 24.82	640.32 ± 58.44 *	447.03 ± 38.63	622.64 ± 40.95 **
Bone volume (mm^3^)	58.30 ± 11.65	70.28 ± 4.81	57.29 ± 12.13	66.35 ± 11.20

* p < 0.05 versus control group after treatment. ** *p* < 0.01 versus control after treatment.
